# Performance Comparison of ChatGPT-4 and Japanese Medical Residents in the General Medicine In-Training Examination: Comparison Study

**DOI:** 10.2196/52202

**Published:** 2023-12-06

**Authors:** Takashi Watari, Soshi Takagi, Kota Sakaguchi, Yuji Nishizaki, Taro Shimizu, Yu Yamamoto, Yasuharu Tokuda

**Affiliations:** 1 General Medicine Center Shimane University Hospital Izumo Japan; 2 Department of Medicine University of Michigan Medical School Ann Arbor, MI United States; 3 Medicine Service VA Ann Arbor Healthcare System Ann Arbor, MI United States; 4 Faculty of Medicine Shimane University Izuom Japan; 5 Division of Medical Education Juntendo University School of Medicine Tokyo Japan; 6 Department of Diagnostic and Generalist Medicine Dokkyo Medical University Hospital Tochigi Japan; 7 Division of General Medicine, Center for Community Medicine Jichi Medical University Tochigi Japan; 8 Muribushi Okinawa Project for Teaching Hospitals Okinawa Japan

**Keywords:** ChatGPT, artificial intelligence, medical education, clinical training, non-English language, ChatGPT-4, Japan, Japanese, Asia, Asian, exam, examination, exams, examinations, NLP, natural language processing, LLM, language model, language models, performance, response, responses, answer, answers, chatbot, chatbots, conversational agent, conversational agents, reasoning, clinical, GM-ITE, self-assessment, residency programs

## Abstract

**Background:**

The reliability of GPT-4, a state-of-the-art expansive language model specializing in clinical reasoning and medical knowledge, remains largely unverified across non-English languages.

**Objective:**

This study aims to compare fundamental clinical competencies between Japanese residents and GPT-4 by using the General Medicine In-Training Examination (GM-ITE).

**Methods:**

We used the GPT-4 model provided by OpenAI and the GM-ITE examination questions for the years 2020, 2021, and 2022 to conduct a comparative analysis. This analysis focused on evaluating the performance of individuals who were concluding their second year of residency in comparison to that of GPT-4. Given the current abilities of GPT-4, our study included only single-choice exam questions, excluding those involving audio, video, or image data. The assessment included 4 categories: general theory (professionalism and medical interviewing), symptomatology and clinical reasoning, physical examinations and clinical procedures, and specific diseases. Additionally, we categorized the questions into 7 specialty fields and 3 levels of difficulty, which were determined based on residents’ correct response rates.

**Results:**

Upon examination of 137 GM-ITE questions in Japanese, GPT-4 scores were significantly higher than the mean scores of residents (residents: 55.8%, GPT-4: 70.1%; *P*<.001). In terms of specific disciplines, GPT-4 scored 23.5 points higher in the “specific diseases,” 30.9 points higher in “obstetrics and gynecology,” and 26.1 points higher in “internal medicine.” In contrast, GPT-4 scores in “medical interviewing and professionalism,” “general practice,” and “psychiatry” were lower than those of the residents, although this discrepancy was not statistically significant. Upon analyzing scores based on question difficulty, GPT-4 scores were 17.2 points lower for easy problems (*P*=.007) but were 25.4 and 24.4 points higher for normal and difficult problems, respectively (*P*<.001). In year-on-year comparisons, GPT-4 scores were 21.7 and 21.5 points higher in the 2020 (*P*=.01) and 2022 (*P*=.003) examinations, respectively, but only 3.5 points higher in the 2021 examinations (no significant difference).

**Conclusions:**

In the Japanese language, GPT-4 also outperformed the average medical residents in the GM-ITE test, originally designed for them. Specifically, GPT-4 demonstrated a tendency to score higher on difficult questions with low resident correct response rates and those demanding a more comprehensive understanding of diseases. However, GPT-4 scored comparatively lower on questions that residents could readily answer, such as those testing attitudes toward patients and professionalism, as well as those necessitating an understanding of context and communication. These findings highlight the strengths and limitations of artificial intelligence applications in medical education and practice.

## Introduction

### Overview

Generative artificial intelligence (AI), such as ChatGPT, stands at the forefront of large-scale language models (LLMs) capable of simulating humanlike dialogues based on user input [1]. ChatGPT, furnished by OpenAI, represents an evolving natural language processing model envisaged as an invaluable asset for future clinical support and medical education within the health care system [1-3]. To date, ChatGPT has achieved passing grades on the US Certified Public Accountant Exam, Bar Exam, and Medical Licensing Examination [2-5]. However, these accomplishments have been attained exclusively in English, and investigations conducted until 2022 cast doubt on its ability to provide medically reliable responses in non-English languages [6]. On March 14, 2023, OpenAI introduced the latest iteration of LLMs, GPT-4 [7,8]. Touted as more reliable and innovative than its predecessor, GPT-3.5, GPT-4 reportedly shows superior performance in non-English languages, particularly in academic and professional contexts [8,9]. However, the extent of the improvement remains unclear. Given the potential applications of the generative AI system represented by GPT-4 in the Japanese medical landscape, it is imperative to assess the accuracy of its use in Japanese medical terminology. This assessment is especially relevant because Japanese is considered among English natives as one of the most challenging languages to master [10]. Interestingly, it has been suggested that GPT-3.5, the precursor to GPT-4, has achieved passing grades on the Japanese Nursing Licensing examination [11]. In the latest Japanese national medical licensing examination in February 2023, GPT-4 attained passing levels while GPT-3.5 showed that it is not far behind the passing criteria [12]. Nonetheless, it is crucial to recognize that these licensing examinations are designed for candidates who have completed their pregraduate education. Consequently, the performance of GPT-4 in terms of actual clinical knowledge and skills following the mandatory postgraduate clinical residency training in Japan remains unverified. Validating its reliability for clinical reasoning and medical knowledge in non-English languages has substantial international implications as it directly affects patient safety and the overall quality of care [13]. Therefore, in this study, we used the General Medicine In-Training Examination (GM-ITE) [14], an internationally validated examination, to compare the performance of Japanese clinical residents with that of GPT-4 to appraise the performance capability of ChatGPT.

### Postgraduate Clinical Training in Japan

Japan maintains a 2-year postgraduate training curriculum instituted by the Ministry of Health, Labor, and Welfare, in which participating physicians are referred to as residents [15,16]. Although trainees are anticipated to develop foundational clinical acumen and broad knowledge coupled with practical abilities to address diverse clinical scenarios during this training, the developments do not equate to specialized curricula such as primary care in the United States or family medicine in the United Kingdom. It is noteworthy that the specialties within general medicine in Japan include “family physician,” “hospitalist,” and “hospital family physician” [17]. These are differentiated based on 2 primary perspectives: differences in clinical settings (eg, rural areas, clinics, city hospitals, and university hospitals) and the ratio of family medicine practices to internal medicine practices, referred to as the clinical operating system [17].

Within this framework, an overwhelming majority of medical students enroll in a residency program after completing 6 years of medical school (residents retain the autonomy to apply to any residency program, with certain delineated exceptions [15]). This obligatory training period is structured to incorporate a minimum of 24 weeks of internal medicine training; 12 weeks of emergency medicine training; and 4 weeks each for surgery, pediatrics, obstetrics and gynecology, psychiatry, and community medicine training across all residency training programs [15,16]. The remaining portion of the clinical training curriculum is set aside for elective training, granting individuals the flexibility to select from their respective training programs.

### Basic Clinical Proficiency Examination: GM-ITE

The Japan Institute for Advancement of Medical Education Program (JAMEP) developed the GM-ITE as a tool for evaluating the fundamental clinical competencies of Japanese clinical residents. This examination has been successfully validated against international clinical examination standards [14,18].

The GM-ITE primarily aims to quantify the degree to which Japanese residents have amassed knowledge, skills, and problem-solving aptitudes throughout their 2-year mandatory clinical training. Ultimately, the examination results serve as feedback for both residents and institutions, identifying areas of weakness and learning requirements for residents. These findings are instrumental in shaping individualized learning assistance and educational guidance, improving the training program environment, and refining residents’ educational plans. Presently, the GM-ITE is implemented as a computer-based test based on the yearly conclusion for postgraduate year (PGY) 1 and PGY2. The examination encompasses multiple-choice questions (60-80 questions) that span a wide array of knowledge and skills in various domains, such as internal medicine, surgery, pediatrics, obstetrics and gynecology, emergency medicine, and psychiatry [14,18]. Over a 3-year period, the cumulative number of questions is 220, with no repeated questions.

## Methods

### Overview

GPT-4 was used to represent the LLM. The exam questions were sourced from the Basic Clinical Proficiency Examination (GM-ITE) administered on January 18-31, 2020; January 17-30, 2021; and January 17-30, 2022 [14]. A total of 11,733 residents in PGY2 participated in these assessments.

### Classification and Difficulty Levels of Exam Questions

The GM-ITE encompasses four categories: (1) medical interviews and professionalism, (2) symptomatology and clinical reasoning, (3) physical examination and clinical procedure, and (4) detailed disease knowledge [14,18].

#### Medical Interview and Professionalism

This section evaluates the candidates’ patient interaction and communication capabilities, comprehension of ethical codes, and professionalism. Questions that are typically scenario-based probe the candidate’s aptitude for conducting appropriate medical interviews, understanding patients, and applying medical ethics.

#### Symptomatology and Clinical Reasoning

This segment measures the ability to discern a diagnosis from history, symptoms, and test results. Candidates are expected to deduce potential diseases from clinical symptoms and patient reports, validate such deductions, and select appropriate treatment options.

#### Physical Examination and Clinical Procedure

This category assesses fundamental physical examination techniques and treatment procedures, along with the ability to interpret such information. The comprehension of the possible diagnoses is also examined.

#### Detailed Disease Knowledge

This section gauges an in-depth understanding of a variety of diseases. The pathophysiology, disease progression, diagnostic methods, and treatment methods are also evaluated. These questions probe a comprehensive understanding of a specific disease and its application to patient care. In this study, questions were categorized into 7 domains (general practice, internal medicine, surgery, pediatrics, obstetrics and gynecology, emergency, and psychiatry) following the standards set by the GM-ITE Examination Preparation Committee. The difficulty level of each question was established based on the percentage of correct answers received by JAMEP. Questions with less than 41.0% correct answers were classified as hard, those with between 41.1% and 72.1% correct answers as normal, and those with more than 72.1% correct answers as easy. The exclusion criteria were questions with images that GPT-4 could not recognize (n=55), questions containing videos (n=22), or both (n=6). The final analysis included 137 questions.

### Data Collection

On July 15-16, 2023, GPT-4 was tasked with answering the aforementioned questions, and the results were subsequently gathered. Each question was inputted once, and the answer was determined. The “correct” answers, as stipulated by JAMEP, served as the reference for comparison. Answers were deemed “correct” only if they explicitly complied with the instructions within the question text. Ambiguous responses that contained blatant errors or contained multiple choices were classified as incorrect. The GM-ITE questions and their multiple-choice options were verbatim, as per the official rubric provided by JAMEP in its original Japanese form. A representative rubric is as follows: “This section presents questions from the Basic Clinical Competency Assessment Test for Initial Residents in Japan. There are five options from *a* to *e*. Please select one of the options that is appropriate for the question.”

### Data Analysis

Using standard descriptive statistics, we calculated various metrics for each data set, including the number, proportion, mean, SD, 95% CI, median, and IQR. A 1-sample proportion test was used to compare the performance of residents with that of GPT-4 in terms of the correct response rate. All tests were 2-tailed, and statistical significance was set at *P*<.05. All analyses were performed using the Stata statistical software (Stata Corp 2015; Stata 17 Base Reference Manual).

### Ethical Considerations

This study was approved by the Ethical Review Committee of JAMEP (Number 23-3) and Shimane University Ethical Review Committee (20230623-3). All participants provided informed consent before participating in the study, following the Declaration of Helsinki and Strengthening the Reporting of Observational Studies in Epidemiology statement guidelines.

## Results

In total, 137 questions from the GM-ITE were used in this study. The results indicated that the overall score percentage of GPT-4 was notably higher than that of Japanese residents (residents: 55.8%, 95% CI 52.1%-59.5%; GPT-4: 70.1%, 95% CI 62.4%-77.7%; *P*<.001)**.**

Table 1 presents the original categories used in this study. The divergence between the 2 groups is presented across the following four areas: (1) medical interviews and professionalism, (2) symptomatology and clinical reasoning, (3) physical examination and clinical procedure, and (4) detailed disease knowledge. Overall, the GPT-4 score was significantly higher than the mean score for residents by 14.3 points (*P*<.001). In particular, the GPT-4 score was 23.5 points higher than the trainee score in the category of “delayed disease knowledge” (*P*<.001). Conversely, in the “medical interview and professionalism” category, which falls under essential knowledge, the GPT-4 score was 8.6 points lower than the average resident score, although this difference was not statistically significant.

Table 2 presents the results of the same comparison across 7 medical domains. The greatest difference (a gain of 30.9 points for the GPT-4 score) was noted in obstetrics and gynecology (*P*=.02), followed by an increase of 26.1 points in internal medicine (*P*<.001). However, the GPT-4 scores were lower than the average resident scores in general practice (–8.6 points) and psychiatry (–7.1 points), although neither of these differences achieved statistical significance.

Table 3 presents a comparison between the 2 groups based on the question difficulty. For “Easy” questions, the ChatGPT-4 score was 17.3 points lower than the mean resident score (*P*=.007). However, for “Normal” and “Hard” questions, the ChatGPT-4 scores were 25.2 and 24.8 points higher, respectively, than the mean resident scores (both *P*<.001).

Table 4 compares the differences between the 2 groups by year (2020, 2021, and 2022). The mean correct response percentage for residents was approximately 53.0%-56.4% on the 3-year exam. Notably, for the 2020 and 2022 GM-ITE questions, GPT-4 scored 21.7 (*P*=.01) and 21.5 (*P*=.003) points higher, respectively, than did residents. However, for the 2021 GM-ITE questions, the GPT-4 score was only 3.5 points higher than the residents’ score (no significant difference).

**Table 1:** Comparison of the scores achieved by GPT-4 and Japanese medical residents across various GM-ITE^a^ categories.

Category	Questions, n (%)	Examinees, % (95% CI)	GPT-4, % (95% CI)	Differences	*P* value
Total	137 (100.0)	55.8 (52.1-59.5)	70.1 (62.4-77.7)	14.3	<.001^b^
Medical interview and professionalism	19 (13.8)	71.8 (61.0-82.6)	63.2 (41.5-84.8)	–8.6	.40
Symptomatology and clinical reasoning	12 (8.8)	47.0 (30.6-63.4)	50.0 (21.7-78.3)	3.0	.84
Physical examination and clinical procedure	36 (26.3)	57.3 (49.3-65.3)	69.4 (54.4-84.5)	12.1	.14
Detailed disease knowledge	70 (51.1)	52.2 (47.9-56.6)	75.7 (65.7-85.8)	23.5	<.001^b^

^a^GM-ITE: General Medicine In-Training Examination.

^b^Statistically significant.

**Table 2:** Comparison of the scores achieved by GPT-4 and Japanese medical residents across various clinical fields (N=137).

Fields	Questions, n (%)	Examinees, % (95% CI)	GPT-4, % (95% CI)	Differences	*P* value
General practice	19 (13.9)	71.8 (61.0-82.6)	63.2 (41.5-84.8)	–8.6	.40
Internal medicine	48 (35.0)	55.2 (49.4-60.9)	81.3 (70.2-92.3)	26.1	<.001^a^
Surgery	9 (6.6)	57.6 (41.3-74.0)	77.8 (50.6-105)	20.2	.22
Pediatrics	12 (8.8)	55.1 (39.6-70.5)	66.7 (40.0-93.3)	11.6	.42
Obstetrics and gynecology	15 (10.9)	49.1 (38.8-59.4)	80.0 (59.6-100)	30.9	.02^a^
Emergency	19 (13.8)	48.1 (37.7-58.5)	57.9 (35.7-80.1)	9.8	.39
Psychiatry	15 (10.9)	53.8 (40.4-67.2)	46.7 (21.4-71.9)	–7.1	.58

^a^Statistically significant.

**Table 3:** Comparison of the scores achieved by GPT-4 and Japanese medical residents across various difficulty levels (N=137).

Difficulty level	Questions, n (%)	Examinees, % (95% CI)	GPT-4, % (95% CI)	Differences	*P* value
Easy	35 (25.6)	82.9 (80.4-85.5)	65.7 (50.0-81.4)	–17.2	.007^a^
Normal	67 (48.9)	56.7 (54.4-59.0)	82.1 (72.9-91.3)	25.4	<.001^a^
Hard	35 (25.6)	27.0 (23.6-30.4)	51.4 (34.9-68.0)	24.4	.001^a^

^a^Statistically significant.

**Table 4:** GPT-4 scores on GM-ITE^a^ by year (N=137).

Year	Questions, n (%)	Examinees, % (95% CI)	GPT-4, % (95% CI)	Differences	*P* value
2020	33 (24.1)	53.9 (46.6-61.1)	75.6 (61.1-90.4)	21.7	.01^b^
2021	56 (40.9)	57.2 (50.8-63.6)	60.7 (47.9-73.5)	3.5	.59
2022	48 (35.0)	55.6 (50.8-63.6)	77.1 (65.2-89.0)	21.5	.003^b^

^a^GM-ITE: General Medicine In-Training Examination.

^b^Statistically significant.

## Discussion

### Principal Findings

This study evaluated the performance of OpenAI ChatGPT-4 on the GM-ITE, an essential Japanese clinical competency test. The findings revealed that the GPT-4 scores surpassed the average scores of residents just before completing their 2-year training period. Furthermore, GPT-4 demonstrated remarkable proficiency in the detailed disease knowledge section, which requires an in-depth understanding of diseases, as well as in more challenging questions and domains, such as internal medicine and obstetrics and gynecology. However, GPT-4 seemed to struggle with questions in the “medical interview and professionalism” and “psychiatry” categories, which are typically easier for residents. A conceivable explanation is that, within the medical domain, examinations primarily serve to authenticate basic comprehension, frequently deviating from genuine patient-focused clinical environments. Such deviations might be more pronounced for LLMs, which are proficient in rapidly integrating available information. Their less-than-optimal results in general practice and psychiatry can be linked to the inherent empirical and intuitive characteristics of these specialties, emphasizing patient-specific context and experiential wisdom over textbook summaries. This nuance is possible because such queries often entail understanding physician roles and making context-sensitive decisions, which are elements deeply rooted in human emotions and experiential nuances. These are dimensions AI cannot yet emulate accurately. The following discussion focuses on the areas where GPT-4 exhibits strengths and weaknesses in handling clinical problems, as well as its performance and advancement in non-English languages.

The superior performance of GPT-4 in the “detailed disease knowledge” category and its adeptness in handling more challenging questions can be attributed to its proficiency in managing detailed knowledge-based queries [19]. AI’s capacity to learn from vast data sets, potentially surpassing the cumulative knowledge of humans, has been highlighted in various studies [19,20]. Consequently, LLMs, such as GPT-4, are expected to excel in scenarios demanding substantial knowledge accumulation, information organization, and recall of specific details that may be difficult for humans to retain [21].

First, in this study, the difficult questions, particularly those related to internal medicine and obstetrics and gynecology, frequently demanded the recall of disease information as well as diagnosis and treatment options. For residents, knowledge pertaining to complex diseases encountered during initial clinical training might be vague because of insufficient exposure. Consistently, prior research on the Japanese national medical examinations found that the performance gap between AI and humans widened with increasing question difficulty [12]. Indeed, AI models such as GPT-4 have achieved the proficiency level required to pass even highly challenging certification examinations that often pose challenges for many humans [2-5,11,12]. Because common clinical scenarios often follow a distinct framework or pattern, AI’s rule-based responses have the potential to surpass human performance [22,23].

However, GPT-4 scored lower on questions in areas such as medical interviewing/professionalism and psychiatry, which demand situational understanding and judgments based on human emotions and experience. Although 1 study noted that ChatGPT expressed more empathy toward patients than physicians [24], AI’s current capability to understand and recognize human emotions remains limited. Therefore, it is reasonable to assume that residents outperform GPT-4 in addressing queries demanding contextual understanding [25]. Considering the structure of the residency training program, the lower performance of medical residents in “internal medicine” and “obstetrics and gynecology” could be attributed to the breadth of these subjects. It is challenging to cover all aspects of these fields during the 24-week and 4-week training periods. Additionally, leveraging AI to solve and analyze clinical evaluation tests could be instrumental in the development of more efficient training programs. By focusing on areas where the AI deviates from the expected responses, we might also be able to evaluate and enhance the validity of the test questions.

Third, the challenges faced by languages other than English should be considered. The majority of the model’s training data consist of English texts, potentially leading to disparate performance levels when dealing with other languages. Comprehending diverse local and sociocultural contexts worldwide is a complex task, and the lack of culturally specific knowledge as well as up-to-date medical literature and data in other languages represents significant limitations for ChatGPT. These limitations may lead to irrelevant or incorrect responses and conclusions [19,26]. In essence, underperformance in non-English languages, particularly concerning its application in health care and medical education, could further exacerbate historical disparities in medical research [27]. Nevertheless, OpenAI’s reports indicate that GPT-4 has demonstrated superior proficiency across 24 out of the 26 assessed languages compared with its predecessor [20]. Although OpenAI does not disclose the exact methodology used to derive these results, the outcomes of this study, which used Japanese, one of the languages most distant from English and difficult for native English speakers to learn, lend credence to OpenAI’s claims [10,20].

### Limitations and Strengths

This study has several limitations. First, the constraints of GPT-4 necessitated the exclusion of examination questions that incorporate images and videos. The GM-ITE is designed to assess basic clinical skills and frequently uses visual information, such as heart sounds, echo videos, computed tomography scans, and electrocardiograms, to reflect actual clinical scenarios more accurately (excluded questions represent 37.7% of all questions in this study). Therefore, we could not thoroughly contrast the competencies of the residents with GPT-4’s performance in decision-making based on visual data. It is essential to emphasize that, within this scope, GPT-4’s potential is somewhat limited, especially when applied to clinical domains that necessitate robust processing and interpretation of visual information. Second, the absence of an interactive format could have deprived GPT-4 of its strengths. One of the key advantages of GPT-4 is its adaptability to clinical scenarios [28]; however, the research method, which uses only multiple-choice questions in a specific format, limits its adaptability. Real-life medical practice requires more advanced clinical reasoning and judgment in interpreting and making sense of chronological information rather than simple cross-sectional knowledge questions. To truly compare the clinical competency of GPT-4 with that of physicians, it is essential to incorporate more practical scenarios into the question design. Third, the performance of GPT-4 may vary over time, and data drift is a major concern [29]. These language models are trained on large data sets, and their performance may degrade if the data distribution changes as time progresses. For example, if a language model is trained using data from a specific period, its performance may deteriorate when exposed to more recent information. Although the data collection window in this study spanned only a few days, making substantial changes improbable, it remains imperative to consistently bear in mind this issue when using continuously evolving generative AI systems [30].

Despite these limitations, this study is the first to demonstrate that GPT-4 outperforms physicians near the end of their mandatory clinical training in the Japanese national exam, the Basic Clinical Competencies Assessment Test. This finding suggests that GPT-4 has potential for application in the medical field, where it can provide information at par with or surpass that offered by novice Japanese trainees. However, further research is required to apply generative AI to non-English languages in both medical practice and education. The gradual accumulation of evidence, clarification of strengths and weaknesses, and incorporation of measures for safety and quality improvements in health care are all essential facets demanding consideration.

### Conclusions

GPT-4 outperformed the average medical residents on the Japanese GM-ITE examination. Notably, GPT-4 scored higher on difficult questions, those with lower correct response rates for residents, and those requiring detailed disease knowledge. Conversely, GPT-4 scored lower on questions requiring patient-centric attitudes and professionalism and those demanding comprehension of context and communication areas in which residents were more proficient. These results compellingly indicate the evolution and utility of AI tools in medical pedagogy and clinical practice. Nevertheless, additional investigations are imperative regarding its potential hazards and security.

## Abbreviations

AI: artificial intelligence
GM-ITE: General Medicine In-Training Examination
JAMEP: Japan Institute for Advancement of Medical Education Program
LLM: large language model
PGY: postgraduate year

## Data Availability

The data supporting the findings of this study are available from the corresponding author upon request.
